# Supplementary material for: Subcellular reorganization upon phage infection reveals stepwise assembly of viral particles from membrane-associated precursors
Source: Nat Commun. 2026 Apr 2;17:4711. doi: 10.1038/s41467-026-71181-w (PMC13212656; doi:10.1038/s41467-026-71181-w)
Supplement: Supplementary file 1 — Supplementary Information [file 41467_2026_71181_MOESM1_ESM.pdf]

# Supplementary Information

## Subcellular reorganization upon phage infection reveals stepwise assembly of viral particles from membrane-associated precursors

*Simon Corroyer-Dulmont, Audrey Labarde, Vojtěch Pražák, Lia M. Godinho, Chloé Masson, Pierre Legrand, Kay Grünewald, Paulo Tavares, Emmanuelle R.J. Quemin*

- Supplementary Figures 1 to 9
- Supplementary Tables 1 to 3

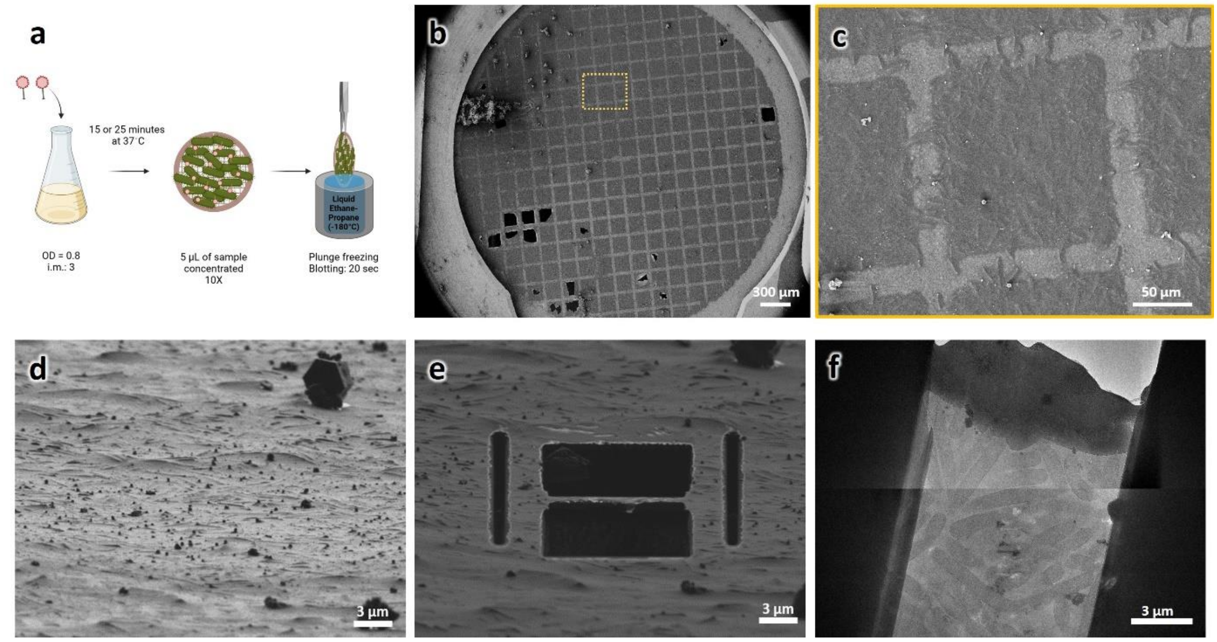


**Supplementary Fig. 1 | Sample preparation workflow developed for studying SPP1-infected bacteria by electron cryo tomography. a.** Schematic representation of the optimized protocol used for sample preparation for cryoET. Following *B. subtilis* infection by SPP1, cells concentrated 10-fold are applied on grids and plunge-frozen in cryogen cooled down to liquid nitrogen temperature. OD, optical density; i.m., input multiplicity. Created with BioRender. **b.** Overview of a sample plunge-frozen on grid observed under cryo conditions in a scanning electron microscope (SEM) equipped with a focused ion beam (FIB), a cryo-FIB-SEM. The yellow rectangle on the SEM view indicates the region enlarged in (**c**) showing a grid square with a continuous layer of *B. subtilis* cells on top. **d,e.** FIB view of the targeted grid area before (**d**) and after FIB-milling (**e**) using the cryo-FIB-SEM to remove material at the top and bottom of the area of interest to make lamellae that are ~100 nm thin. **f.** Overview of a representative lamella containing cross-sections of several *B. subtilis* cells observed after transfer to a Titan Krios for cryoET data collection. Scale bars are indicated.

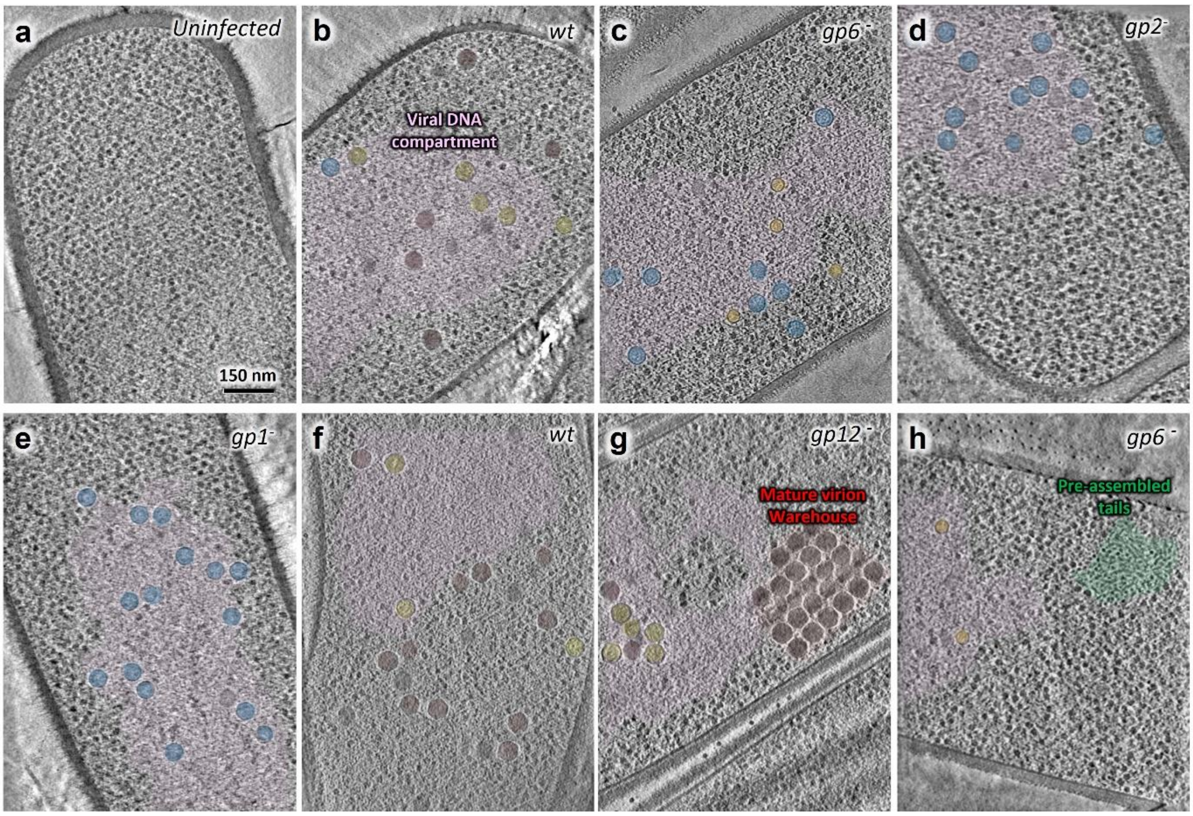


**Supplementary Fig. 2 | Overview of the vDNA compartment and viral particle structures observed in bacteria infected by wild type and mutant SPP1 phages. a.** Slice from a representative tomogram of uninfected *B. subtilis* cell (shown in Fig. 1a; Supplementary Movie 1). **b.** Slice from a representative tomogram of *B. subtilis* infected by SPP1 wild type (*wt*; shown in Fig. 1b; Supplementary Movie 2) with the vDNA compartment visible (pink) containing different assembly intermediates: procapsids I (blue), procapsids II (yellow), and DNA-filled capsids (red). **c.** Slice from a representative tomogram of *B. subtilis* infected by SPP1*gp6*$^-$ (*gp6*$^-$; shown in Fig. 3c; Supplementary Movie 5) with the vDNA compartment visible (pink) containing procapsids I-like structures (blue) and small procapsid-like structures (orange). **d.** Slice from a representative tomogram of *B. subtilis* infected by SPP1*lacO64gp2*$^-$ (*gp2*$^-$; shown in Fig. 4a; Supplementary Movie 6) with the vDNA compartment visible (pink) containing exclusively procapsids I (blue). **e.** Slice from a representative tomogram of *B. subtilis* infected by SPP1*gp1*$^-$ (*gp1*$^-$) with the vDNA compartment visible (pink) containing exclusively procapsids I (blue). **f.** Slice from a representative tomogram of *B. subtilis* infected by SPP1 wild type (*wt*; shown in Fig. 5a; Supplementary Movie 7) with the vDNA compartment visible (pink) and a diffuse warehouse at its periphery containing mature virions (red). **g.** Slice from a representative tomogram of *B. subtilis* infected by SPP1*lacO64gp12*$^-$ (*gp12*$^-$; shown in 5b; Supplementary Movie 8) with the vDNA compartment visible (pink) and a packed warehouse at its periphery containing mature virions (red). **h.** Slice from a representative tomogram of *B. subtilis* infected by SPP1*gp6*$^-$ (*gp6*$^-$; shown in Fig. 5c; Supplementary Movie 9) with the vDNA compartment visible (pink) as well as preassembled tails (green) clustered at its periphery. Scale bars are indicated.

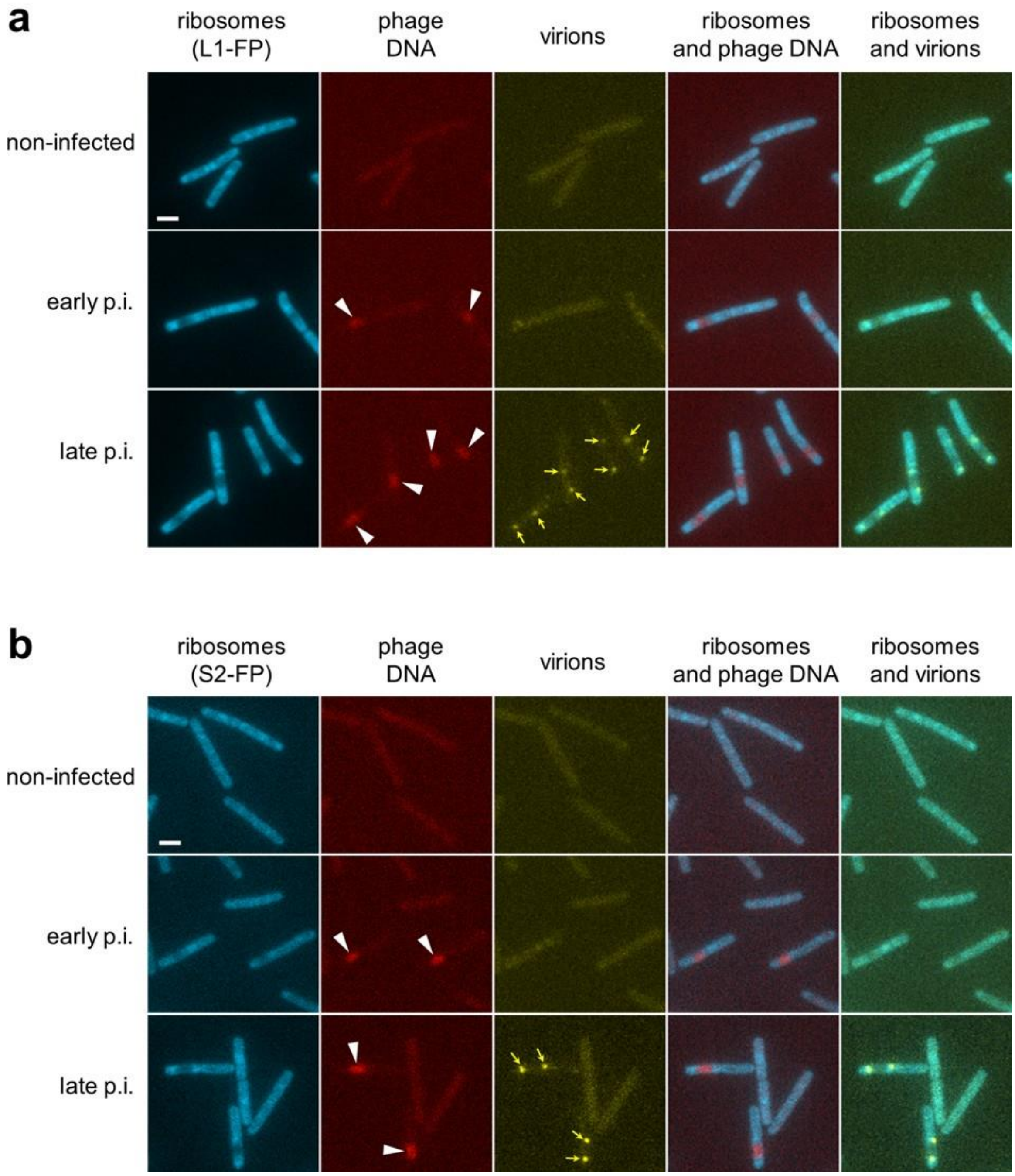


**Supplementary Fig. 3 | Localization of ribosomes, SPP1 DNA and virions inside the reorganized bacterial cells.** Ribosomes are viewed by imaging ribosomal proteins L1 (or RplA in *B. subtilis*) (**a**) and S2 (or RpsB in *B. subtilis*) (**b**) fused to CFP (see Methods). Bacteria infected by SPP1*lacO64gp12*$^-$ were imaged at 30 min p.i. (early p.i.) and 55 min p.i. (late p.i.). Phage DNA is visualized with LacI-mCherry to define the vDNA compartment highlighted by white arrowheads in the RFP channel images. Virions are visualized with gp12-mCitrine and their warehouses highlighted by yellow arrows in the YFP channel images. Scale bars represent 2 µm. Results of experiments displayed are representative of two independent biological replicates.

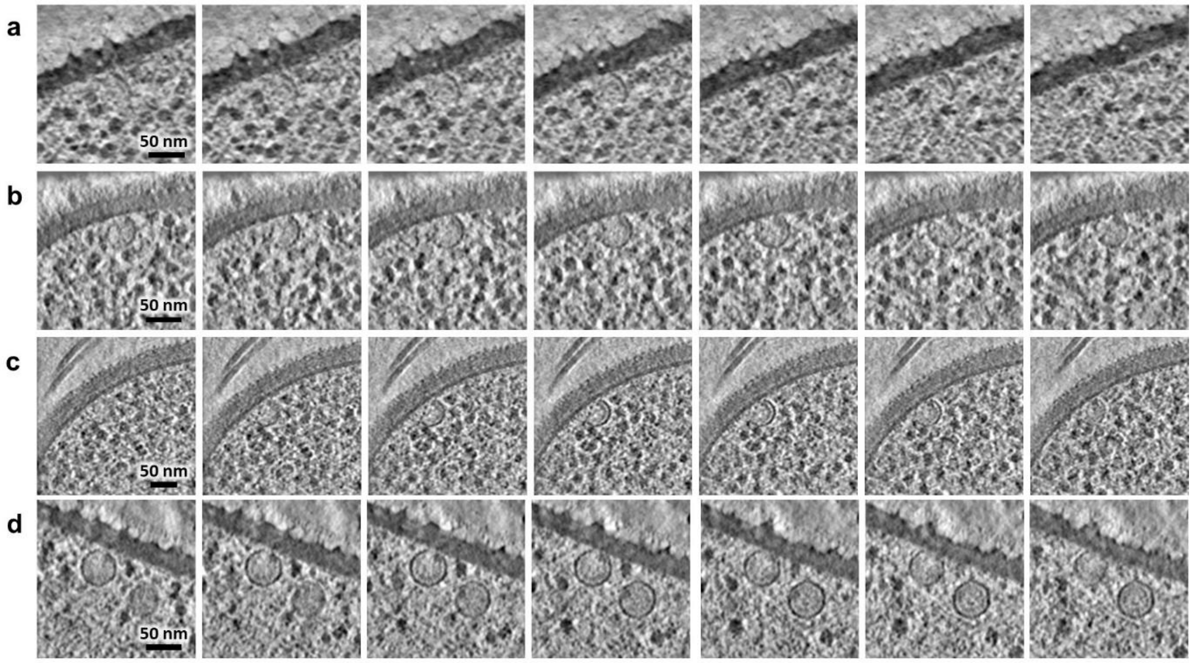


**Supplementary Fig. 4 | Procapsid assembly precursors.** Gallery of slices through representative tomograms of *B. subtilis* infected by SPP1*wt*. Intermediates at different stages of procapsid assembly are showed at the bacterial cell membrane from an initial stage (**a**) to a complete procapsid I-like particle associated to the inner side of the bacterial cell membrane or detached (**d**). Each precursor of assembly intermediates is depicted here at different z heights while only a central slice is shown in Fig. 2. Z step: 2.6 nm for (**a,b**) and 3.4 nm for (**c,d**). Scale bars are indicated.

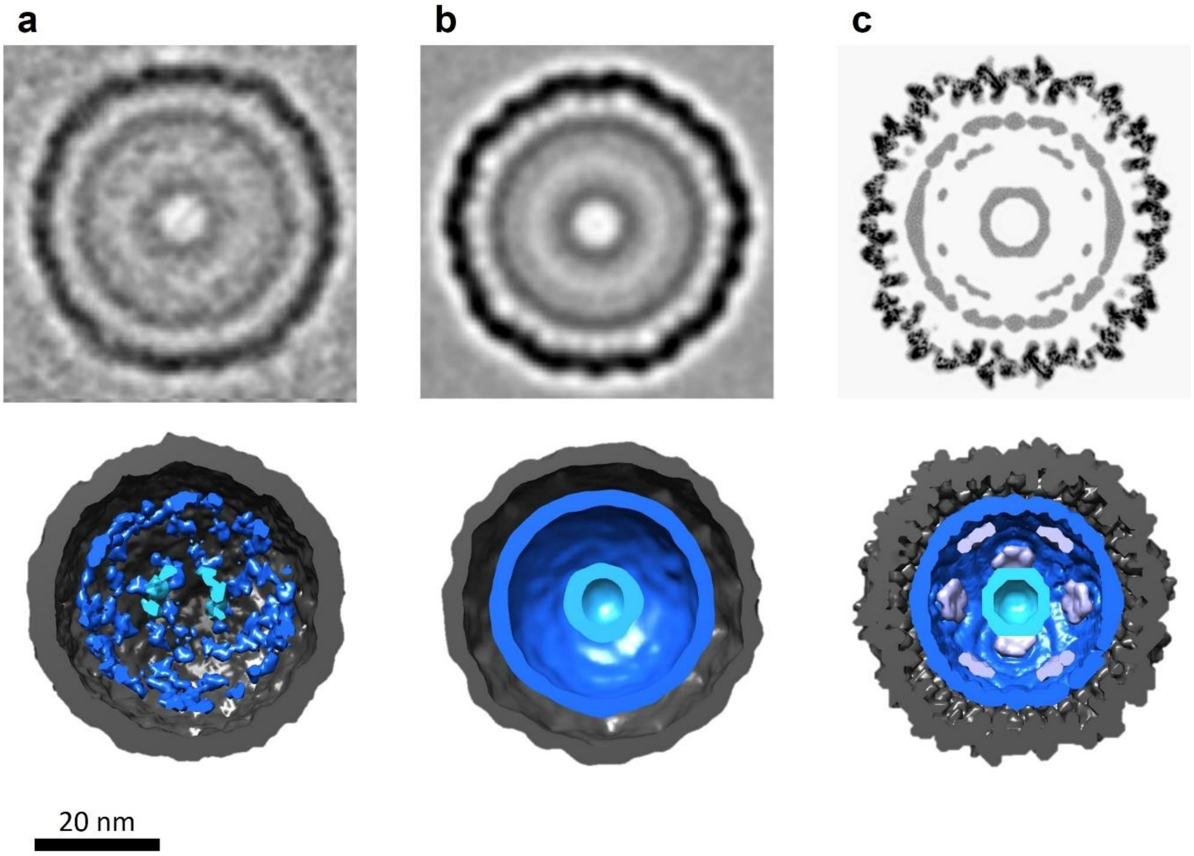


**Supplementary Fig. 5 | Sub-volume averaging and comparison of procapsid I structures found *in cellula* and *in vitro*.** Gallery of slices through averages of procapsids I from a dataset *in cellula* of SPP1*wt*, SPP1*lacO64gp1*⁻ and SPP1*lacO64gp2*⁻ infections, accompanied by their respective volume rendering (bottom), compared to a cryoEM structure of *in vitro* purified particles published previously (EMD-4717) [1]. (**a**) Average using one model point per capsid located at its centre (n = 572). (**b**) Average after applying icosahedral symmetry using model points located at the centre of the capsid's pentons (n = 6864). (**c**) EM map from purified procapsid I *in vitro* determined by cryoEM single-particle analysis (EMD-4717)[1]. Densities of features of interest are displayed with the inner shell in light blue and outer shell in dark blue, corresponding to the scaffolding protein, as well as intermediate densities in purple and the capsid in black. The scale bar indicates 20 nm.

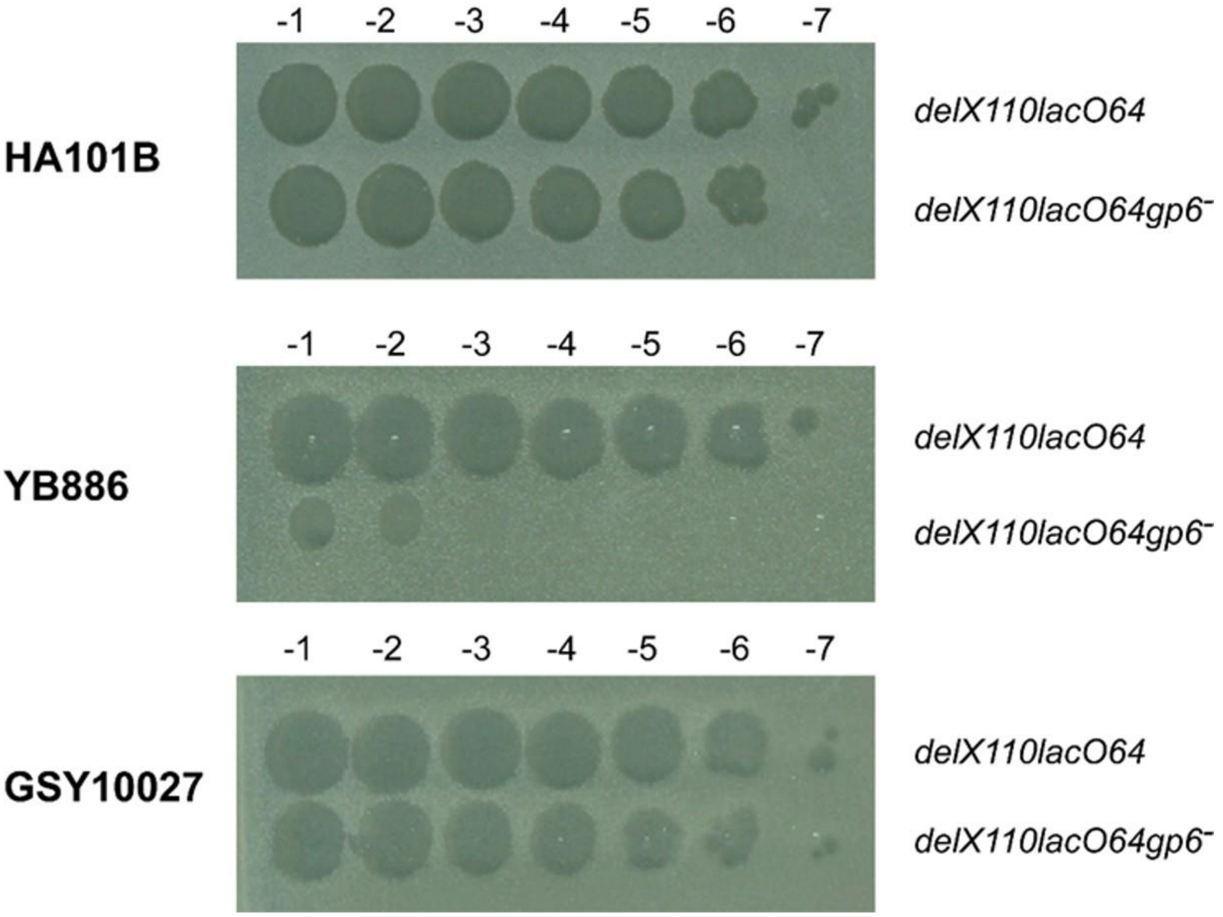


**Supplementary Fig. 6 | Trans-complementation of SPP1*gp6*⁻ with gp6-mCitrine *in vivo*.** Infection of *B. subtilis* permissive strain HA101B, non-permissive strain YB886, and strain GSY10027 by the suppressor-sensitive mutant SPP1*lacO64gp6*⁻ and by the control phage SPP1*lacO64* (*wt*). GSY10027 is an YB886-derived strain that encodes gp6-mCitrine (see Methods). A 2 µL spot of each phage serial dilution was deposited on top of the indicator bacteria lawns. Phage stocks were initially diluted to $10^8$ pfu.mL$^{-1}$ for serial dilutions from $10^{-1}$ to $10^{-7}$ as indicated on top.

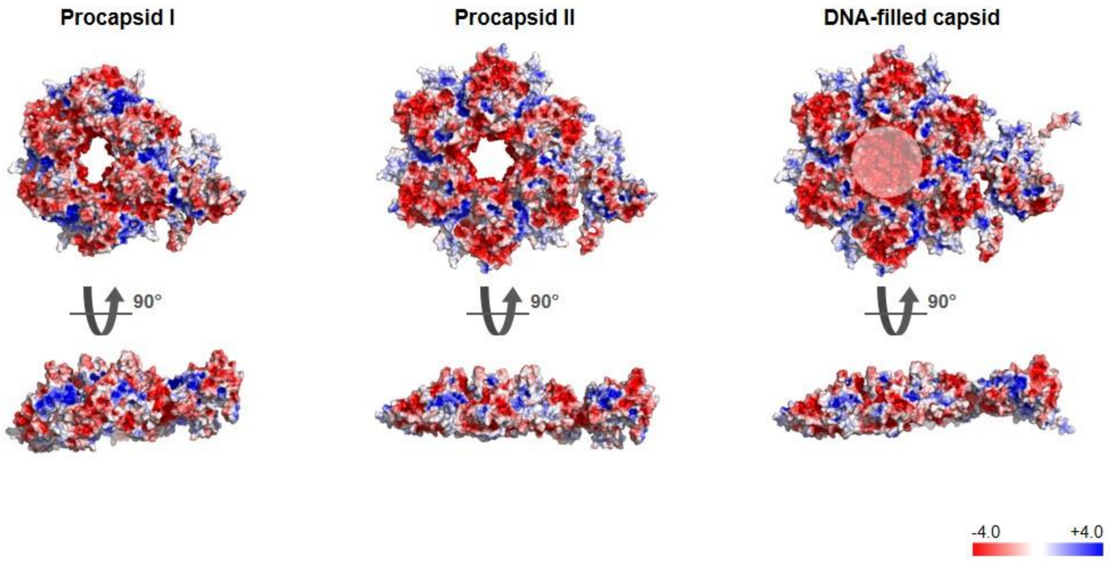


**Supplementary Fig. 7 | Surface electrostatic potential of the asymmetric units of procapsid I, procapsid II and DNA-filled capsid.** Local electrostatic potential values are displayed on views from the structure exterior (top) and turned 90° (bottom). Accession numbers for the PDB coordinates of the atomic models are 6R3B, 6RTL, and 6R3A, respectively[1]. Electrostatic potentials calculated with PyMOL are rendered in red and blue from -4.0 kT/e (red) to 4.0 kT/e (blue); as indicated on the scale bar on the bottom right side. The semi-transparent circle in the center of the hexon represents the accessory protein gp12 whose chain was not traced in the DNA-filled capsid structure[1].

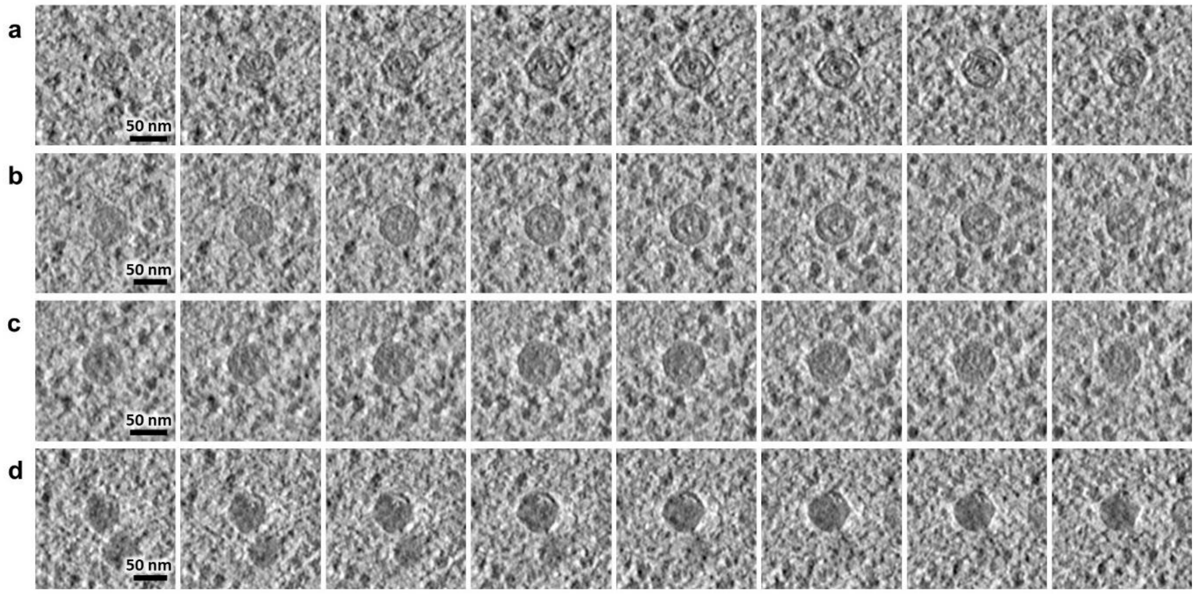


**Supplementary Fig. 8 | Procapsids II at different stages of viral genome encapsidation.** Gallery of slices through representative tomograms of *B. subtilis* infected by SPP1*wt*. The gallery displays several procapsids II from an initial stage of genome packaging with a low amount of DNA observed inside the procapsid (**a**) to an almost filled capsid (**d**) and intermediates (**b** and **c**). Individual procapsids II are depicted here at different z heights to highlight how the viral genome is arranged on the inside during the process of encapsidation. Z step is 3.4 nm and scale bars are indicated.

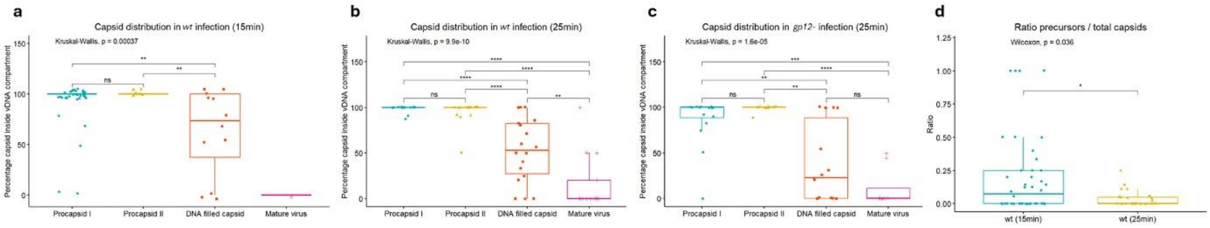


**Supplementary Fig. 9 | Statistical analyses on the timing and localization of the viral assembly intermediates in SPP1-infected cells. a-c**. Percentage of the different assembly intermediates (procapsids I, procapsids II, DNA-filled capsids and mature virions) found inside the vDNA compartment at 15 min p.i. (**a**) and 25 min p.i. (**b**) in SPP1*wt* infection or at 25 min p.i. for SPP1*gp12*- infection (**c**). Regarding the distribution, the boundaries of the vDNA compartment were arbitrarily defined by the exclusion of the ribosomes (as exemplified in Fig. 1 and in Supplementary Fig. 2). **d**. Ratio of precursors compared to the total number of capsids (procapsids I, procapsids II, DNA-filled capsids and mature virions) is plotted for each tomogram. Precursors are considered as incomplete procapsid structures found at the bacterial cell membrane as in Fig. 2 and Supplementary Fig. 4. Statistically-relevant differences were determined using either a Kruskal-Wallis test (non-parametric one-way ANOVA test) followed by a pairwise, two-sided, Wilcoxon post-hoc test, or an unpaired, two-sided Wilcoxon test (non-parametric t-Student test). Statistical significance is indicated by star symbols and assessed by a *p*-value below 0.05 (*), below 0.01 (**), below 0.001 (***), or below 0.0001 (****); ns, not significant. Each dot in the box plot corresponds to one tomogram analysed: n = 50 (SPP1*wt* at 15 min p.i.), n=20 (SPP1*wt* at 25 min p.i.), n=18 (SPP1*lacO64gp12*$^-$ at 25min p.i.). The horizontal line represents the median value for each condition. Source data are provided as Source Data file 2.

**Supplementary Table 1. Quantification of capsid structures observed in wild type and mutant SPP1 infection conditions. a.** Number of capsid assembly intermediates and their location into the compartmentalized host bacterial cell observed in the tomograms under the different conditions of infection. The localisation inside the infected host bacterial cell have been divided as either "at the cell membrane" for precursors found at the membrane or "inside" and "outside" the vDNA compartment for the other stages of assembly observed. **b.** Percentage of the capsid assembly intermediates found specifically in the vDNA compartment in the different infection conditions analysed. **c.** Percentage of capsid assembly intermediates observed in the different infection conditions compared to the total number of events.

**a. Number of capsid assembly intermediates observed in the different infection conditions either at the membrane, inside or outside of the vDNA compartment**

| | Procapsid precursor | Small procapsid I | | Procapsid I | | Procapsid II | | DNA-filled capsid | | Mature virus with tail | | Total number of particles | Number of tomograms |
|---|---|---|---|---|---|---|---|---|---|---|---|---|---|
| | At membrane | Inside | Outside | Inside | Outside | Inside | Outside | Inside | Outside | Inside | Outside | | |
| *gp1*$^{-}$ | 2 | 0 | 0 | 358 | 0 | 0 | 0 | 0 | 0 | 0 | 0 | 360 | 9 |
| *gp2*$^{-}$ | 10 | 0 | 0 | 467 | 5 | 0 | 0 | 0 | 0 | 0 | 0 | 482 | 28 |
| *gp12*$^{-}$ | 15 | 0 | 0 | 129 | 17 | 85 | 10 | 225 | 199 | 6 | 42 | 728 | 25 |
| *gp6*$^{-}$ (15 min) | 0 | 17 | 18 | 22 | 2 | 0 | 0 | 0 | 0 | 0 | 0 | 59 | 29 |
| *gp6*$^{-}$ (25 min) | 0 | 150 | 12 | 246 | 10 | 0 | 0 | 0 | 0 | 0 | 0 | 418 | 29 |
| *wt* (15 min) | 28 | 0 | 0 | 126 | 6 | 19 | 0 | 18 | 7 | 0 | 1 | 205 | 50 |
| *wt* (25 min) | 12 | 0 | 0 | 87 | 3 | 107 | 4 | 120 | 35 | 12 | 52 | 432 | 25 |

**b. Percentage of capsid assembly intermediate found in the vDNA compartment per infection condition**

| | Small procapsid I | Procapsid I | Procapsid II | DNA-filled capsid | Mature virus with tail |
|---|---|---|---|---|---|
| *gp1*$^{-}$ | 0% | 100% | 0% | 0% | 0% |
| *gp2*$^{-}$ | 0% | 99% | 0% | 0% | 0% |
| *gp12*$^{-}$ | 0% | 88% | 89% | 53% | 13% |
| *gp6*$^{-}$ (15 min) | 49% | 92% | 0% | 0% | 0% |
| *gp6*$^{-}$ (25 min) | 93% | 96% | 0% | 0% | 0% |
| *wt* (15 min) | 0% | 95% | 100% | 72% | 0% |
| *wt* (25 min) | 0% | 97% | 96% | 77% | 19% |

**c. Percentage of capsid assembly intermediates observed in the different infection conditions**

| | Procapsid precursor | Small procapsid I | Procapsid I | Procapsid II | DNA-filled capsid | Mature virus with tail |
|---|---|---|---|---|---|---|
| *gp1*$^{-}$ | 0.5% | 0% | 99.5% | 0% | 0% | 0% |
| *gp2*$^{-}$ | 2.1% | 0% | 97.9% | 0% | 0% | 0% |
| *gp12*$^{-}$ | 2.1% | 0% | 20.1% | 13% | 58.2% | 6.6% |
| *gp6*$^{-}$ (15 min) | 0% | 59.3% | 40.7% | 0% | 0% | 0% |
| *gp6*$^{-}$ (25 min) | 0% | 38.8% | 61.2% | 0% | 0% | 0% |
| *wt* (15 min) | 13.7% | 0% | 64.4% | 9.3% | 12.2% | 0.4% |
| *wt* (25 min) | 2.8% | 0% | 20.8% | 25.7% | 35.9% | 14.8% |

**Supplementary Table 2. Bacterial strains, viral strains, plasmids and oligonucleotides used in this work.** List of biological resources including bacterial strains, bacteriophage strains, plasmids and sequence of oligonucleotides (restriction sites used for cloning are in bold) employed in this work together with description of the source and short names used in the text. Their use is described in Methods.

| BIOLOGICAL RESOURCES | SOURCE | SHORT NAME |
|---|---|---|
| **Bacterial strains** | | |
| *E. coli F- Φ80lacZΔM15 Δ(lacZYA-argF) U169 recA1 endA1 hsdR17 (rk-, mk+) phoA supE44 λ-thi-1 gyrA96 relA1* | Hanahan, 1983[2] | DH5α |
| *B. subtilis amyE trpC2 metB5 xin-1 attSPβ* | Yasbin et al., 1980[3] | YB886 |
| *B. subtilis [his met]* $sup^-$ *leu trpC2* | Okubo and Yanagida, 1968[4] | HA101B |
| *B. subtilis amyE trpC2 metB5 xin-1 attSPβ (pSPW7)* | Tavares et al., 1992[5] | YB886 (pSPW7) |
| *B. subtilis amyE trpC2 metB5 xin-1 attSPβ thrC::(*$P_{pen}$*-lacIΔ11-mcherry mls)* | Labarde et al., 2021[6] | GSY10004 |
| *B. subtilis amyE trpC2 metB5 xin-1 attSPβ thrC::(*$P_{pen}$*-lacIΔ11-mcherry mls) sacA::(Pspac-12-mcitrine kan)* | Labarde et al., 2021[6] | GSY10024 |
| *B. subtilis amyE trpC2 metB5 xin-1 attSPβ thrC::(*$P_{pen}$*-lacIΔ11-mcherry mls) sacA::(*$P_{spac}$*-6-mcitrine kan)* | This work | GSY10027 |
| *B. subtilis amyE trpC2 metB5 xin-1 attSPβ amyE::(*$P_{xyl}$*-rpsB-mcfp spc)* | This work | GSY10074 |
| *B. subtilis amyE trpC2 metB5 xin-1 attSPβ thrC::(*$P_{pen}$*-lacIΔ11-mcherry mls) amyE::(*$P_{xyl}$*-rpsB-mcfp spc)* | This work | GSY10075 |
| *B. subtilis amyE trpC2 metB5 xin-1 attSPβ thrC::(*$P_{pen}$*-lacIΔ11-mcherry mls) sacA::(*$P_{spac}$*-12-mcitrine kan) amyE::(*$P_{xyl}$*-rpsB-mcfp spc)* | This work | GSY10082 |
| *B. subtilis amyE trpC2 metB5 xin-1 attSPβ amyE::(*$P_{xyl}$*-rplA-mcfp spc)* | This work | GSY10085 |
| *B. subtilis amyE trpC2 metB5 xin-1 attSPβ thrC::(Ppen-lacIΔ11-mcherry mls) amyE ::(*$P_{xyl}$*-rplA-mcfp spc)* | This work | GSY10086 |
| *B. subtilis amyE trpC2 metB5 xin-1 attSPβ thrC::(Ppen-lacIΔ11-mcherry mls) sacA::(*$P_{spac}$*-12-mcitrine kan) amyE::(*$P_{xyl}$*-rplA-mcfp spc)* | This work | GSY10087 |
| **Bacteriophage strains** | | |
| SPP1*sus70*: phage SPP1 defective in gene *1*. Used for cryoET experiments. | Behrens et al., 1979[7]; Chai et al., 1992[8] | SPP1$gp1^-$ ($gp1^-$) |
| SPP1*sus115*: phage SPP1 defective in gene *6*. Used for cryoET experiments. | Behrens et al., 1979[7]; Tavares et al 1992[5] | SPP1$gp6^-$ ($gp6^-$) |

| | | |
|---|---|---|
| SPP1*delX110lacO64*: phage SPP1 carrying deletion *delX* and an array of *lacO* operators. Used in this work for productive, wild type infections. | Jakutyte et al., 2011[9] | SPP1*lacO64* (or *wt*) |
| SPP1*delX110lacO64sus19*: Phage SPP1 carrying deletion *delX*, an array of *lacO* operators, and defective in gene *2*. | Labarde et al., 2021[6] | SPP1*lacO64gp2*$^-$ (or *gp2*$^-$) |
| SPP1*delX110lacO64sus115* phage SPP1 carrying deletion *delX*, an array of *lacO* operators and defective in gene *6*. Used for TIRF experiments. | This work | SPP1*lacO64gp6*$^-$ |
| SPP1*delX110lacO64Δ12*: Phage SPP1 carrying deletion *delX*, an array of *lacO* operators and deletion *Δ12*. | Labarde et al., 2021[6] | SPP1*lacO64gp12*$^-$ (or *gp12*$^-$) |
| **Plasmids** | | |
| pSPW7 | Tavares et al., 1992[5] | NA |
| pAL21 *bla sacA::(Pspac-mcitrine kan)* | Labarde et al., 2021[6] | NA |
| pAL27 *bla sacA::(Pspac-6-mcitrine kan)* | This work | NA |
| pAL29 *bla amyE::(Pxyl-mcfp spc)* | Labarde et al., 2021[6] | NA |
| pLG50 *bla amyE::(Pxyl-rpla-mcfp spc)* | This work | NA |
| pLG51 *bla amyE::(Pxyl-rpsb-mcfp spc)* | This work | NA |
| **Oligonucleotides** | | |
| 2677 rev: gggtgcctctctgtg | Lab collection | 2677 |
| 3185 rev: ctctggcggccacctctcgc | Lab collection | 3185 |
| 3383 fwd: gtgtccgaattgcggcccgg | Lab collection | 3383 |
| 3682 fwd: gagttgtgtggatatccaagg | Lab collection | 3682 |
| rplA_KpnI_fwd : taaccacataagga**ggtacc**tttaaaatggctaa | This work | 20 |
| rpsB_KpnI_fwd : ttagga**ggtacc**aacatgtcagtcatt | This work | 22 |
| rplA_EcoRI_rev : gg**gaattc**gccaccttttacgttaaaagttgaagagtc | This work | 28 |
| rpsB_XhoI_rev : cctttgaat**ctcgag**cgcagttgttgt | This work | 29 |
| gp6_ClaI_fwd : ggtaccgagctcgaattcaaaggagaa**atcgat**atggctgatatctacccactaggg | This work | 55 |
| gp6_XhoI_rev : gcttaccattccactgccttcgag**ctcgag**agatacttgtccagctcctcc | This work | 56 |

**Supplementary Table 3. Sample preparation and data collection parameters for electron cryo tomography.** List of samples with the number of tilt-series collected in parenthesis. Then information for sample preparation on grid by vitrification with the LEICA EM GP is given together with the parameters used for data collection for all datasets reported in this study.

| | **Dataset 1** | **Dataset 2** | **Dataset 3** |
|---|---|---|---|
| **Samples with number of tilt-series analysed** | - Uninfected (9)<br>- Infection with SPP1*gp12*$^-$ (9) | - Uninfected (18)<br>- Infection with SPP1*wt* (75)<br>- Infection with SPP1*gp1*$^-$ (9)<br>- Infection with SPP1*gp2*$^-$ (28)<br>- Infection with SPP1*gp6*$^-$ (58) | - Infection with SPP1*wt* (9)<br>- Infection with SPP1*gp12*$^-$ (16) |
| Grid type | Quantifoil® R2/2 Cu 200 | | |
| Grid glow discharge | 30 sec - 15 mA (Pelco easiGlow™) | | |
| Vitrification | Leica EM GP | | |
| Sample Volume | 4 µL | | |
| Sample concentration | $10^9$ cells/mL | | |
| Blotting position | 44 and 4.4 | | |
| Blot paper | Whatman 1 | | |
| Blot time | 20 sec | | |
| Temperature | 22 °C | | |
| Relative humidity | 80 % | | |
| Wait time | 0 sec | | |
| Cryogen | Ethane | | |
| Microscope | Titan Krios G3 | | |
| Voltage | 300 kV | | |
| Energy filter / Detector | Gatan BioQuantum and K3 | | |
| Stilt width | 20 eV | | |
| Objective aperture | 70 µm | | |
| Nominal Magnification | 26,000X | 42,000X | 64,000X |
| Pixel size | 3.336 Å | 2.15 Å | 1.372 Å |
| Defocus range | -4 to -5 µm | | |
| Acquisition Scheme | Dose-symmetric tilt scheme:<br>+/- 60 degrees with 3 degree increment steps | | |
| Dose per frame | 5e-/Å² over 3 frames | 4.5 e-/Å² over 5 frames | 2.5e-/Å² over 2 frames |
| Total dose | ~200 e-/Å² | ~180 e-/Å² | ~120 e-/Å² |

## Supplementary References


1. Ignatiou, A. *et al.* Structural transitions during the scaffolding-driven assembly of a viral capsid. *Nat. Commun.* **10**, 4840 (2019). DOI: 10.1038/s41467-019-12790-6

2. Hanahan, D. Studies on Transformation of *Escherichia coli* with Plasmids. J. Mol. Biol. 166, 557–580 (1983). DOI: 10.1016/s0022-2836(83)80284-8

3. Yasbin R. E., Fields P.I., Andersen B.J. Properties of *Bacillus subtilis* 168 derivatives freed of their natural prophages. Gene 12, 155–159 (1980). DOI: 10.1016/0378-1119(80)90026-8

4. Okubo S., Yanagida T., Isolation of a suppressible mutant in *Bacillus subtilis*. J Bacteriol 95, 1187–1188 (1968). DOI: 10.1128/jb.95.3.1187-1188.1968

5. Tavares P., *et al*. Identification of a gene in *Bacillus subtilis* bacteriophage SPP1 determining the amount of packaged DNA. J. Mol. Biol. 225, 81–92 (1992). DOI: 10.1016/0022-2836(92)91027-m

6. Labarde A. *et al.* Compartmentalization of viral infection in bacterial cells. Proc. Natl. Acad. Sci. USA 118, e2018297118 (2021). DOI: 10.1073/pnas.2018297118

7. Behrens B., Lüder G., Behncke M., Trautner T. A. The Genome of *B. subtilis* Phage SPP1: Physical Arrangement of Phage genes. MGG Mol. Gen. Genet. 175, 351–357 (1979). DOI: 10.1007/BF00397235

8. Chai S., *et al.* Molecular Analysis of the *Bacillus subtilis* Bacteriophage SPP1 Region Encompassing Genes 1 to 6 SPP1 - The Products of Gene 1 and Gene 2 are Required for pac Cleavage. J. Mol. Biol. 224, 87–102 (1992). DOI: 10.1016/0022-2836(92)90578-8

9. Jakutyte L., *et al.* Bacteriophage infection in rod-shaped Gram-positive bacteria: Evidence for a preferential polar route for phage SPP1 entry in *Bacillus subtilis*. J. Bacteriol. 193, 4893–4903 (2011). DOI: 10.1128/JB.05104-11
