# Supplementary material for: Subcellular reorganization upon phage infection reveals stepwise assembly of viral particles from membrane-associated precursors
Source: Nat Commun. 2026 Apr 2;17:4711. doi: 10.1038/s41467-026-71181-w (PMC13212656; doi:10.1038/s41467-026-71181-w)
Supplement: Supplementary file 2 — Description Of Additional Supplementary File [file 41467_2026_71181_MOESM2_ESM.pdf]

Description of Additional supplementary files

Title: Supplementary Movie 1 | Representative tomogram of uninfected *B. subtilis*.

Description: The tomogram displays the typical cellular organization observed with the cytoplasm full of ribosomes except for a small area that contains the bacterial DNA. Segmentation rendering of this tomogram is illustrated in Fig. 1a and an original slice is showed in Supplementary Fig. 2a.

Title: Supplementary Movie 2 | Representative tomogram of *B. subtilis* infected with SPP1*wt*.

Description: The tomogram exhibits the typical host cell re-organization observed upon SPP1*wt* infection with a large area containing viral DNA and some capsid assembly intermediates with a clear exclusion of ribosomes. Segmentation rendering of this tomogram is illustrated in Fig. 1b and an original slice is showed in Supplementary Fig. 2b with colour overlay.

Title: Supplementary Movie 3| TIRF microscopy and time-lapse imaging of gp6-mCitrine in non-infected *B. subtilis*.

Description: *B. subtilis* cells producing gp6-mCitrine were imaged under TIRF illumination for 1 min with an acquisition every 2 s. Gp6-mCitrine small foci found in the perimembrane region are highly mobile. Snapshots of the movie are shown on the right panels of Fig. 3a.

Title: Supplementary Movie 4 | TIRF microscopy and time-lapse imaging of gp6-mCitrine in *B. subtilis* infected with SPP1*lacO64gp6*$^-$.

Description: *B. subtilis* cells producing gp6-mCitrine were infected with SPP1*lacO64gp6*$^-$ for 23 min at 37°C and imaged under TIRF illumination for 1 min with an acquisition every 2 s. Most gp6-mCitrine gets recruited to immobile intense foci localized outside the viral DNA compartment. Snapshots of the movie are shown on the right panels of Fig. 3b.

Title: Supplementary Movie 5 | Representative tomogram of *B. subtilis* infected with SPP1*gp6*$^{-}$.

Description: The tomogram exhibits the typical host cell re-organization observed upon infection with phage SPP1*gp6*$^{-}$ that is defective in production of the portal protein. Procapsid I-like structures and

small procapsids I are found in the viral DNA compartment while aberrant capsid-like structures are visible at its periphery. Segmentation rendering of this tomogram is illustrated in Fig. 3c and an original slice is showed in Supplementary Fig. 2c with colour overlay.

Title: Supplementary Movie 6 | Representative tomogram of *B. subtilis* infected with SPP1*lacO64gp2*$^{-}$.

Description: The tomogram exhibits the typical host cell re-organization observed upon infection with SPP1*lacO64gp2*$^{-}$ that is defective in viral DNA packaging. Only procapsids I are found in the viral DNA compartment while one procapsid precursor is visible at the cell membrane. Segmentation rendering of this tomogram is illustrated in Fig. 4a and an original slice is showed in Supplementary Fig. 2d with colour overlay.

Title: Supplementary Movie 7 | Representative tomogram of mature virions warehouse in *B. subtilis* infected with SPP1 *wt*.

Description: The tomogram exhibits the typical host cell re-organization observed upon infection with SPP1 *wt*. Mature virions are found as clusters at the periphery of the viral DNA compartment. Segmentation rendering of this tomogram is illustrated in Fig. 5a and an original slice is showed in Supplementary Fig. 2f with colour overlay.

Title: Supplementary Movie 8 | Representative tomogram of mature virions warehouse in *B. subtilis* infected with SPP1*lacO64gp12*$^{-}$.

Description: The tomogram exhibits the typical host cell re-organization observed upon infection with SPP1*lacO64gp12*$^{-}$. Mature virions lacking the auxiliary capsid protein gp12, are densely packed at the periphery of the viral DNA compartment in so-called warehouses under this infection condition. Segmentation rendering of this tomogram is illustrated in Fig. 5b and an original slice is showed in Supplementary Fig. 2g with colour overlay.

Title: Supplementary Movie 9 | Representative tomogram of mature virions warehouse in *B. subtilis* infected with SPP1*gp6*$^{-}$.

Description: The tomogram exhibits the typical host cell re-organization observed upon infection with SPP1*gp6*$^{-}$. Portal-less procapsid I-like structures defective in DNA packaging and free tails accumulate in these cells. Indeed, in this condition of infection, aggregates of pre-assembled tail are found in the cytoplasm. Segmentation rendering of this tomogram is illustrated in Fig. 5c and an original slice is showed in Supplementary Fig. 2h with colour overlay.
