# Supplementary material for: Subcellular reorganization upon phage infection reveals stepwise assembly of viral particles from membrane-associated precursors
Source: Nat Commun. 2026 Apr 2;17:4711. doi: 10.1038/s41467-026-71181-w (PMC13212656; doi:10.1038/s41467-026-71181-w)
Supplement: Supplementary file 12 — Reporting summary [file 41467_2026_71181_MOESM12_ESM.pdf]

nature portfolio

Corresponding author(s): Paulo Tavares and Emmanuelle Quemin

Last updated by author(s): Feb 16, 2026

Reporting Summary

Nature Portfolio wishes to improve the reproducibility of the work that we publish. This form provides structure for consistency and transparency in reporting. For further information on Nature Portfolio policies, see our Editorial Policies and the Editorial Policy Checklist.

Statistics

For all statistical analyses, confirm that the following items are present in the figure legend, table legend, main text, or Methods section.

n/a	Confirmed	
☐	☒	The exact sample size (*n*) for each experimental group/condition, given as a discrete number and unit of measurement
☐	☒	A statement on whether measurements were taken from distinct samples or whether the same sample was measured repeatedly
☐	☒	The statistical test(s) used AND whether they are one- or two-sided<br>*Only common tests should be described solely by name; describe more complex techniques in the Methods section.*
☒	☐	A description of all covariates tested
☐	☒	A description of any assumptions or corrections, such as tests of normality and adjustment for multiple comparisons
☐	☒	A full description of the statistical parameters including central tendency (e.g. means) or other basic estimates (e.g. regression coefficient) AND variation (e.g. standard deviation) or associated estimates of uncertainty (e.g. confidence intervals)
☐	☒	For null hypothesis testing, the test statistic (e.g. *F*, *t*, *r*) with confidence intervals, effect sizes, degrees of freedom and *P* value noted<br>*Give P values as exact values whenever suitable.*
☒	☐	For Bayesian analysis, information on the choice of priors and Markov chain Monte Carlo settings
☒	☐	For hierarchical and complex designs, identification of the appropriate level for tests and full reporting of outcomes
☒	☐	Estimates of effect sizes (e.g. Cohen's *d*, Pearson's *r*), indicating how they were calculated

Our web collection on statistics for biologists contains articles on many of the points above.

Software and code

Policy information about availability of computer code

Data collection	SerialEM
Data analysis	IMOD v4.12.40, Aretomo, ISONET, PEET v1.14.1, Chimera, TEMPy, PyMOL

For manuscripts utilizing custom algorithms or software that are central to the research but not yet described in published literature, software must be made available to editors and reviewers. We strongly encourage code deposition in a community repository (e.g. GitHub). See the Nature Portfolio guidelines for submitting code & software for further information.

Data

Policy information about availability of data

All manuscripts must include a data availability statement. This statement should provide the following information, where applicable:

- Accession codes, unique identifiers, or web links for publicly available datasets
- A description of any restrictions on data availability
- For clinical datasets or third party data, please ensure that the statement adheres to our policy

Average map of the procapsid I included in this paper has been deposited in the Electron Microscopy 640 Data Bank (EMDB) with the following accession code: EMD-54016
Previous published accession codes of structures quoted in the manuscript and availability of all data are reported in the manuscript "Data Availability" section

Research involving human participants, their data, or biological material

Policy information about studies with human participants or human data. See also policy information about sex, gender (identity/presentation), and sexual orientation and race, ethnicity and racism.

Reporting on sex and gender	n.a.
Reporting on race, ethnicity, or other socially relevant groupings	n.a.
Population characteristics	n.a.
Recruitment	n.a.
Ethics oversight	n.a.

Note that full information on the approval of the study protocol must also be provided in the manuscript.

Field-specific reporting

Please select the one below that is the best fit for your research. If you are not sure, read the appropriate sections before making your selection.

☒ Life sciences ☐ Behavioural & social sciences ☐ Ecological, evolutionary & environmental sciences

For a reference copy of the document with all sections, see nature.com/documents/nr-reporting-summary-flat.pdf

Life sciences study design

All studies must disclose on these points even when the disclosure is negative.

Sample size	CryoET:<br>Samples with number of tilt-series collected:<br>Dataset 1: Uninfected (9); Infection with SPP1gp12- (9)<br>Dataset 2: Uninfected (18); Infection with SPP1wt (75); Infection with SPP1gp1- (9); Infection with SPP1gp2- 28); Infection with SPP1gp6- (58)<br>Dataset 3: Infection with SPP1wt (9); Infection with SPP1gp12- (16)<br><br>Fluorescence microscopy:<br>TIRF time-lapse experiments of gp6-mCitrine were repeated three times independently with similar results. A total of 23 movies of non-infected bacteria (a) and 50 movies of infected bacteria (b) were recorded.<br>Co-imaging of Bacillus subtilis ribosomes with phage DNA and viral particles was carried out with two reporter ribosomal proteins (RplA and RpsB) in two independent experiments showing a highly reproducible ribosome localization phenotype at early and late times post-infection.The reproducible localization of phage DNA and viral particles is similar to results of a previous study (Labarde et al 2021; doi: 10.1073/pnas.2018297118).
Data exclusions	Tilt-series with poor quality of alignment during reconstruction were excluded from the analysis. For sub-volume averaging in particular, those not containing the object of interest were not taken into acount for this type of analysis as described in the text.
Replication	described in the manuscript
Randomization	n.a. the study did not involve clinical trials, randomization did not apply for this study
Blinding	n.a. blinding is used in clinical trials thus not applicable here

Reporting for specific materials, systems and methods

We require information from authors about some types of materials, experimental systems and methods used in many studies. Here, indicate whether each material, system or method listed is relevant to your study. If you are not sure if a list item applies to your research, read the appropriate section before selecting a response.

Materials & experimental systems

n/a	Involved in the study
☒	☐ Antibodies
☒	☐ Eukaryotic cell lines
☒	☐ Palaeontology and archaeology
☒	☐ Animals and other organisms
☒	☐ Clinical data
☒	☐ Dual use research of concern
☒	☐ Plants

Methods

n/a	Involved in the study
☒	☐ ChIP-seq
☒	☐ Flow cytometry
☒	☐ MRI-based neuroimaging

Plants

Seed stocks	n.a.
Novel plant genotypes	n.a.
Authentication	n.a.
